# Isolated Nasopharyngeal Castleman Disease: An Uncommon Diagnosis in an Unusual Location

**DOI:** 10.1155/2014/475690

**Published:** 2014-07-14

**Authors:** O. McDonnell, Melinda Morris, Z. Khaleel

**Affiliations:** NIISWA, Sir Charles Gairdner Hospital, Hospital Avenue, Nedlands, Perth, WA 6009, Australia

## Abstract

Localised nasopharyngeal Castleman disease has rarely been reported. We present a case involving a 23-year-old female, describe the clinical, imaging, and histopathologic features of this challenging diagnosis, and review the literature.

## 1. Introduction

Castleman disease (CD) is an uncommon benign lymphoproliferative disorder that usually is found in the mediastinum. Castleman disease limited to the mucosa of the upper aerodigestive tract is exceedingly rare. We, herein, report the clinical, MRI, and pathological findings of localised nasopharyngeal Castleman disease in a 23-year-old female and provide a brief review of the disorder focusing on the hyaline vascular subtype. Castleman disease is a challenging diagnosis. Preoperatively, lymphoma or minor salivary gland tumour was the chief differential diagnoses in this case.

## 2. Case Presentation

A 23-year-old female presented with nasal obstruction since childhood. She had symptoms of obstructive sleep apnoea with snoring and daytime somnolence. She did not have constitutional symptoms. Initial fibreoptic nasal endoscopy examination revealed a mass arising from the posterior nasal space and filling the nasopharynx.

**Figure 1:**
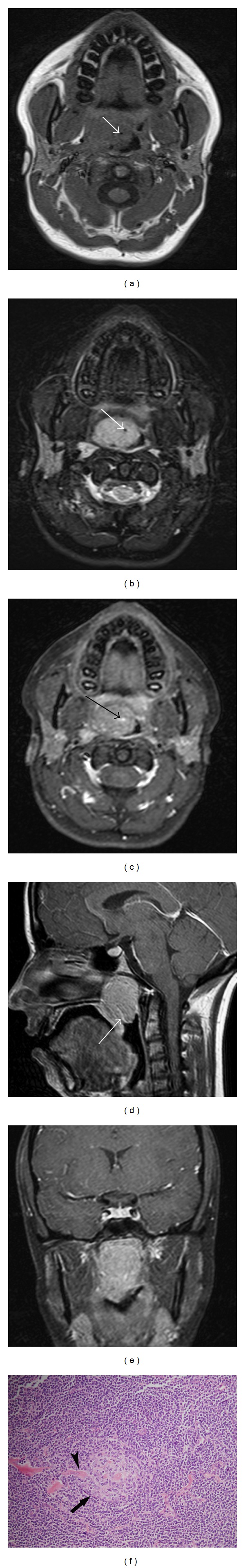
MRI scan demonstrates a homogeneous mucosally based mass occupying almost the entire nasopharyngeal airway extending from the posterior margin of the nasal septum anteriorly to the fossae of Rosenmuller posteriorly. The mass is isointense on unenhanced T1-weighted image (a) and hyperintense on T2-weighted image (b) and shows moderate contrast enhancement (c, d, and e). A hypointense curvilinear flow void that enhances avidly representing the feeding vessel is in the inferior aspect of the tumour (white arrows). (f) Photomicrograph (original magnification ×200) haematoxylin-eosin stain shows lymphoid follicle (black arrow) with atrophic germinal centre. The follicle is rimmed by a concentric layer of small lymphocytes. The germinal centre is penetrated by hyalinised vessels (black arrow head) and there is an increase in interfollicular vasculature. Reactive small lymphocytes are present in the interfollicular regions. The features are consistent with hyaline vascular Castleman's disease.

MRI demonstrated a large mucosally based mass in the nasopharynx (Figures 1(a)–1(e)). Based on the imaging findings, the diagnoses of lymphoma versus minor salivary gland carcinoma were considered. The patient subsequently underwent endoscopically assisted excision of the mass.

Histopathologic features were consistent with hyaline vascular CD. Lymphoid follicles with germinal centres were seen just below the epithelium. Some germinal centres were penetrated by hyalinised vessels and there was an increase in interfollicular vasculature (Figure 1(f)). Flow cytometry and IgH gene rearrangement studies revealed a polyclonal reactive population of lymphocytes.

The patient has remained well and asymptomatic at 2-year followup.

## 3. Discussion

Castleman disease (CD) was first described by Dr. Benjamin Castleman in 1956 [1] and is also known as angiofollicular lymph node hyperplasia, giant lymph node hyperplasia, or angiofollicular lymphoid hamartoma. It is an uncommon condition but one of the recognised causes of nonneoplastic lymphadenopathy.

The traditional classification of the disease as unicentric versus multicentric variants is falling out of favor and being replaced by a newer histopathogenic classification. The latter consists of 4 subtypes: hyaline vascular CD, plasma cell CD, human herpes virus 8 (HHV-8) associated CD, and multicentric CD not otherwise specified [2]. Unicentric CD is most frequently the hyaline vascular subtype, and multicentric CD is typically the plasma cell subtype of the disease [2].

The median age of presentation is in the fourth decade [2] although the age range is broad and pediatric cases have been reported. There is no gender predilection. The disease can occur in almost any area where lymphoid tissue is normally present. The chest is the most common site of involvement (approximately 70%) followed by the neck (15%) and the abdomen (15%) but extra lymphatic sites such as lungs, larynx, parotid glands, pancreas, meninges, and muscles can also occur [3].

Localised nasopharyngeal CD is extremely rare and to our knowledge the present case is only the fourth reported case in the nasopharynx. The first report was by Tsai et al. [4] of a 19-year-old male who presented with nasal obstruction and was found to have a pedunculated nasopharyngeal tumour suspected to be an angiofibroma. Chan et al. [5] described an unusual case of 23-year-old male with nasopharyngeal mass that proved to be CD with focal overgrowth of follicular dendritic cells (FDC), which three years later recurred as FDC sarcoma. The third reported case was by Kim et al. [6] of a 34-year-old female who presented with a pedunculated nasopharyngeal mass.

The differential diagnosis depends on the location of disease and in our case included lymphoma and minor salivary gland tumour. The disease cannot be distinguished by clinical manifestations or imaging analysis alone and excisional biopsy is often required to make a definitive diagnosis. Our case and the three previously reported cases of nasopharyngeal CD had histopathologic features consistent with hyaline vascular CD.

Hyaline vascular CD is the most common (90%) histological subtype. It is unicentric in 90% and is usually asymptomatic with a benign course. Only 3% of patients with localised disease are symptomatic. However, if symptoms occur they almost always relate to compression of adjacent organs. Indeed our patient had symptoms of obstructive sleep apnea. Hyaline vascular CD is often curable with surgical resection.

Microscopically the hyaline vascular variant is characterised by small hyalinised follicular centers and prominent interfollicular vascular proliferation with perivascular hyalinisation. The hyalinised centers are surrounded by sheets of lymphocytes that give them an “onion skin” appearance. Often, a single penetrating vessel is seen in the center of the follicle. Plasmacytoid dendritic cell collections may be found to be associated with these lesions [2].

The aetiology of hyaline vascular CD remains unclear, but some relationship has been reported with dysplastic follicular dendritic cells [7]. In addition, vascular proliferation in this subtype might be related to vascular endothelial growth factor [8].

Both CT and MRI help to define the extent of Castleman disease. In the nasopharynx, the disease is better demonstrated with MRI. MRI findings are of homogeneous contrast enhancing masses with the hyaline vascular variant showing more enhancement than the other subtypes. Most CD masses are isointense or mildly hyperintense on T1-weighted images and hyperintense on T2-weighted images when compared with muscle [9]. Occasionally, arborising linear hypointense signals can be detected within the lesion, as in our case, which are thought to represent the flow voids of the feeding vessels [9] acting as an important clue to the diagnosis. This finding is predominantly observed in the hyaline vascular variant [3].

The prognosis for localised hyaline vascular CD is excellent and our patient has remained disease free two years following tumour resection.

Although an association with follicular dendritic cancer has been reported [5], this is exceedingly rare. Follicular dendritic cell sarcoma is an uncommon tumour with a low to intermediate risk of recurrence or metastasis.

## 4. Conclusion

Isolated nasopharyngeal Castleman disease is an uncommon diagnosis with only three previously reported cases in the literature all of which were of the hyaline vascular variant. It is a challenging diagnosis which requires clinical, imaging, and histopathological correlation and should be considered in the differential diagnosis of a mass in this location.
